# Dietary Macronutrient Composition and Risk of Radiation-Induced Acute Skin Toxicity in Women with Breast Cancer: Results from the ATHENA Project

**DOI:** 10.3390/nu17010136

**Published:** 2024-12-31

**Authors:** Sukshma Sharma, Francesca Bracone, Augusto Di Castelnuovo, Emilia Ruggiero, Amalia De Curtis, Francesco Deodato, Gabriella Macchia, Mariangela Boccardi, Savino Cilla, Alessio Giuseppe Morganti, Chiara Cerletti, Giovanni de Gaetano, Katia Petroni, Chiara Tonelli, Maria Benedetta Donati, Licia Iacoviello, Marialaura Bonaccio, on behalf of the EU-ATHENA Trial Investigators

**Affiliations:** 1 Research Unit of Epidemiology and Prevention, IRCCS NEUROMED, 86077 Pozzilli, Italy; research.sukshma@gmail.com (S.S.); francesca.bracone@moli-sani.org (F.B.); dicastel@moli-sani.org (A.D.C.); emilia.ruggiero@moli-sani.org (E.R.); amalia.decurtis@moli-sani.org (A.D.C.); chiara.cerletti@moli-sani.org (C.C.); giovanni.degaetano@moli-sani.org (G.d.G.); mbdonati@moli-sani.org (M.B.D.); marialaura.bonaccio@moli-sani.org (M.B.); 2 Istituto di Radiologia, Università Cattolica S. Cuore, 00168 Rome, Italy; francesco.deodato@unicatt.it; 3 Radiotherapy Unit, Responsible Research Hospital, 86100 Campobasso, Italy; macchiagabriella@gmail.com (G.M.); mariangela.boccardi@responsible.hospital (M.B.); 4 Medical Physic Unit, Responsible Research Hospital, 86100 Campobasso, Italy; savinocilla@gmail.com; 5 Department of Experimental, Diagnostic and Speciality Medicine—DIMES, Alma Mater Studiorum Bologna University, 40138 Bologna, Italy; alessio.morganti2@unibo.it; 6 Department of Biosciences, Università degli Studi di Milano, 20133 Milano, Italy; katia.petroni@unimi.it (K.P.); chiara.tonelli@unimi.it (C.T.); 7 Department of Medicine and Surgery, LUM University, 70010 Casamassima, Italy

**Keywords:** breast cancer, radiotherapy, skin toxicity, macronutrient, carbohydrate, fat, protein

## Abstract

Background: The impact of the dietary macronutrient composition and its subcomponents (saccharides, fatty acids, and protein sources) on radiation-induced acute skin toxicity (AST) in breast cancer (BC) patients is unknown. Hence, we examined the association between dietary macronutrients and their subcomponents and the risk of ≥grade 2 (G2) AST post-radiotherapy among women with BC. Methods: An observational study was conducted among 161 BC patients treated with radiotherapy and enrolled in the ATHENA project in Italy. Habitual dietary intake was assessed at study entry (T0) using a 188-item food frequency questionnaire (FFQ). AST was measured at T1 (after 3 or 5 weeks of radiotherapy) and defined according to the Radiation Therapy Oncology Group criteria. A prospective analysis used multivariable-adjusted logistic regression models to examine the association between the dietary macronutrient composition and its subcomponents at T0 and the odds of ≥G2 AST post-radiotherapy. Results: ≥G2 AST post-radiotherapy was observed in 43 (27%) patients. Among dietary macronutrient models, a higher intake of dietary carbohydrates was positively associated with a 30% higher odds of ≥G2 AST post-radiotherapy (OR = 1.30; 95% CI 1.01 to 1.67; for 30 g/d). Conversely, a higher dietary protein intake was inversely associated with a 76% lower odds of ≥G2 AST post-radiotherapy (OR = 0.24; 95% CI 0.06 to 0.91; for 30 g/d). There was no association with dietary fat. In macronutrient subcomponent models, only animal protein was inversely associated with a 51% lower odds of ≥G2 AST post-radiotherapy (0.49; 95% CI 0.25 to 0.95; for 15 g/d). Conclusions: Dietary carbohydrates were associated with a higher risk of radiation-induced AST among women with BC, whereas dietary protein, especially animal protein, was associated with a lower risk. Cautiously balancing carbohydrate and protein intakes could be a part of the clinical management strategy for ≥G2 AST reduction post-radiotherapy among BC women.

## 1. Introduction

Breast cancer (BC) is the most commonly diagnosed type of cancer in women, with an estimated 2.3 million new cases per year, thus posing a significant burden on public health [1]. Further, almost 50% of BC cases are estimated to require adjuvant radiotherapy as a post-surgical approach [2,3,4] resulting in different degrees of radiation-induced skin injury. These are of two types: (1) acute skin toxicity (AST) involving dry and wet desquamation and skin ulcers, and (2) chronic skin toxicity changes, including skin fibrosis and moderate telangiectasias [5]. A recent systematic review and meta-analysis [6] of 38 studies composed of 15,623 BC patients reported considerable heterogeneity in the evaluation of AST and in patient- and treatment-related risk factors. The proportion of patients with AST of grade (G) 2 or higher after radiotherapy ranged from 10% to 76%, with an average of 34.3% and a median of 28.4% [6].

Diet is a well-established modifiable risk factor that significantly influences the prevention and management of BC [7,8]. For instance, Western diets and unhealthy dietary patterns high in refined grains, sweets, and high-fat dairy products are associated with a high BC risk [9,10,11]. Meanwhile, prudent and healthy dietary patterns high in vegetables, limited saturated fat intakes, and processed meats are associated with a lower BC risk [8,11]. Further, regarding the dietary macronutrient composition, observational studies suggested that higher carbohydrate and fat intakes were associated with higher BC risk [12,13,14].

However, there is sparse epidemiological evidence on the impact of the dietary macronutrient composition on BC patients receiving radiotherapy and its associated side effects, such as AST. This gap makes providing evidence-based dietary advice for AST in clinical settings challenging, and BC patients often use complementary and alternate medicine (herbs, antioxidants, and vitamins) as potential solutions to improve AST and quality of life after a radiotherapy cycle [15]. So far, high-fiber diets are suggested to lower the risk of AST, but only in patients with pelvic, head, and neck cancers [16]. An animal study suggested that a high-fat diet potentially lowered the risk of AST caused by ionizing radiation in rats [17]. It remains unknown whether the dietary macronutrient composition and their subcomponents (saccharides, fatty acids, and protein sources) differently impact the risk of AST during radiation in BC cases.

Therefore, we aimed to investigate specific associations between dietary macronutrient intakes (carbohydrates, protein, and fats and their subcomponents, such as saccharides, fatty acids, and type of dietary protein) and the risk of ≥grade (G) 2 (moderate/severe) AST among 161 BC patients post-radiotherapy within the ATHENA project.

## 2. Subjects and Methods

### 2.1. Trial Design and Participants

**Figure 1:**
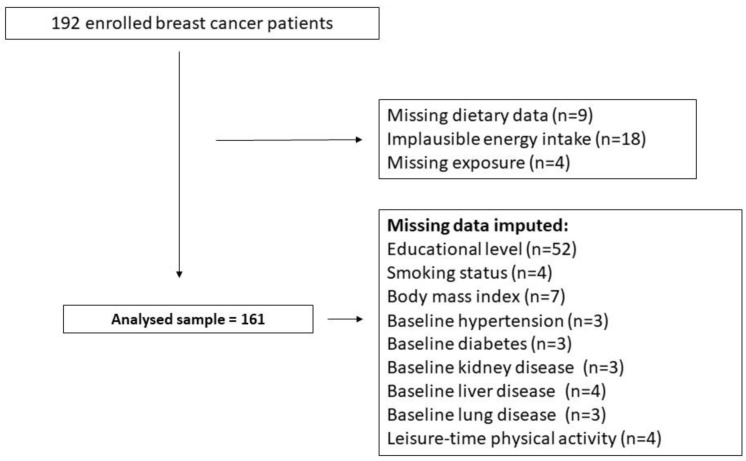
Flow chart of the ATHENA trial.

The current research was an analysis of an independent observational study conducted under the ATHENA project, a double-blind, randomized, placebo-controlled trial to test the effects of purple corn cob-derived-anthocyanin supplementation on skin toxicity among BC patients undergoing radiotherapy [18]. Overall, 161 women with BC who underwent radiotherapy were finally analyzed in the current study. Detailed accounts of the ATHENA project’s randomized clinical trial (RCT) methodology are published elsewhere [18]. A flow chart of the ATHENA trial is shown in Figure 1.

### 2.2. Eligibility Criteria

Participants above 18 years of age, diagnosed with BC, and eligible for radiotherapy were recruited in the study. The medical staff pre-screened participants and recruited them based on the inclusion and exclusion criteria of the ATHENA RCT [18]. All participants with invasive carcinoma of the breast, surgical treatment (lumpectomy and quadrantectomy), and axillary staging were included in the study. Participants who were pregnant or lactating at the time of recruitment, with psychiatric or addictive disorders, or diagnosed non-invasive/synchronous bilateral invasive/non-epithelial breast malignancies, proven multicentric carcinoma, or any other prior breast or thoracic radiotherapy for any conditions were excluded at the recruitment phase [18].

The trial, performed at the Gemelli Molise Hospital Radiotherapy Unit in Campobasso, Italy, was conducted according to the guidelines of the Declaration of Helsinki. The patient recruitment was conducted between 9 June 2014, and 26 June 2017, and the trial concluded on 10 October 2018 (the date of the last patient’s one-year follow-up). The protocol was approved by two ethical committees: Catholic University, Rome, and the Azienda Sanitaria Regionale del Molise (ASReM). All participating patients signed written informed consent, previously approved by the Ethical Committees. The study was registered in clinicaltrials.gov with the identifier ID: NCT02195960. The ATHENA project trial conformed to the Consolidated Standards of Reporting Trials (CONSORT) consortium [19].

### 2.3. Radiotherapy Treatment

The ATHENA project RCT was divided into two groups of patients, according to the risk of recurrence (low or moderate to high). The first group included patients with a low risk (patients underwent a 3-week treatment with hypofractionated radiotherapy, residual breast: 40 Gy (Gray) in 2.5 Gy/fraction; concomitant boost on the tumor bed: 4 Gy in 0.25 Gy/fraction). The second group included patients with a moderate high risk of recurrence (underwent a 5-week treatment with standard doses of radiotherapy, residual breast: 50 Gy in 2 Gy/fraction; concomitant boost on tumor bed: 10 Gy in 0.40 Gy/fraction). All patients (3- and 5-week schedules) were treated with forward-planned intensity-modulated radiation therapy (IMRT). Patients were advised to use a topical cream (Atonderma Radiomed^®^) on the irradiated site around 2–3 hr before and after each session every day from the beginning of radiotherapy [18].

For the present analysis, we considered data from the entire study population that received radiotherapy treatment, without dividing them based on the duration of radiotherapy treatment or the randomized assignment to an anthocyanin-rich treatment.

### 2.4. Dietary Assessment

Participant’s habitual dietary intake was assessed at study entry (T0) by an interviewer-administered semi-quantitative EPIC (European Prospective Investigation into Cancer and Nutrition) food frequency questionnaire (FFQ) validated and adapted to the Italian population [20], to assess the participants’ diet during the preceding 12 months. The FFQ contains 14 sections (i.e., pasta/rice, soup, meat (excluding salami and other cured meats), fish, raw vegetables, cooked vegetables, eggs, sandwiches, salami and other cured meats, cheese, fruit, bread/wine, milk/coffee/cakes, and herbs/spices) with 248 questions concerning 188 different food items [20]. Using specifically designed software, the frequencies and quantities of each food were linked to Italian Food Tables [21] to obtain estimates of the daily intake of macro- and micronutrients plus energy.

The primary exposures were total energy and macronutrients, including carbohydrates, protein, and fat, and their subcomponents, which were recorded at baseline (T0). The carbohydrate subcomponents included complex sugars (starch, dietary fiber, and soluble fiber). The dietary fat subcomponents included saturated fatty acids (SFAs), mono-unsaturated fatty acids (MUFAs), and poly-unsaturated fatty acids (PUFAs). The protein subcomponents included animal and vegetable proteins.

### 2.5. Definition of Study Outcomes

The main outcome of the present study was to evaluate the odds of ≥G2 AST. During the visits following radiotherapy, the medical staff recorded acute skin toxicity according to the Radiation Therapy Oncology Group (RTOG) criteria [22]. AST was measured until 3 or 5 weeks post-radiotherapy (T1) by assessing the skin characteristics at the irradiated site and was defined as follows: “0” for Grade 0, no skin changes, or Grade 1, follicular, faint, or dull erythema/epilation/dry desquamation/decreased sweating; and “1” for Grade 2, tender or bright erythema, patchy moist desquamation/moderate edema; Grade 3, confluent, moist desquamation other than skin folds, pitting edema; and Grade 4, ulceration, hemorrhage, and necrosis [22,23].

### 2.6. Assessment of Covariates

At the baseline visit (T0), each patient’s medical history, anthropometric and clinical measures, and dietary and lifestyle habits were collected. The following factors were included as covariates in the analysis: age (in years), body mass index (calculated as kilograms/meters square), C-reactive protein (CRP) levels at T0, alcohol consumption (0 = no/low and high intake; 1 = moderate moderate), hypertension (systolic blood pressure [SBP] >140 mmHg and/or diastolic [DBP] >90 mmHg or anti-hypertensive treatment), smoking habits (1 = yes, 0 = no or 2 = former), prescribed hormone therapy (letrozole) (1= yes, 0 = no), and treatment classification (B = treatment [anthocyanin supplementation]/A = placebo) recorded at baseline (T0).

The covariates were identified and included in the analysis based on directed acyclic graphs (DAGs) [24,25]. DAGs provide a straightforward and visual presentation for identifying and testing assumptions about causal relationships between variables by deducing an algorithm, thus providing an adjustment set of covariates for estimating causal effects.

### 2.7. Statistical Analyses

Data were represented as numbers and percentages for categorical variables or the mean and standard deviation (SD) for continuous variables.

Multivariable-adjusted logistic regression models (Models 1 and 2) were designed to explore the association between dietary macronutrients or their subcomponents and the percentage of energy (%E) from macronutrients at baseline (T0) and the odds of ≥G2 AST amongst post-radiation BC patients after 3 or 5 weeks of undergoing radiotherapy. Model 1 minimally adjusted for baseline covariates, including age, BMI, and treatment classification (treatment/placebo), and model 2 additionally adjusted for hypertension, alcohol consumption, smoking habits, CRP levels at baseline, and prescribed hormone therapy. Further, each macronutrient and its subcomponent model were adjusted for other energy-contributing macronutrients.

Total energy and dietary macronutrients and their subcomponent data (exposures) were recorded as continuous variables. AST (outcome) was coded as a binary outcome variable (“0” denoted the absence of ≥G2 AST, and “1” denoted the presence of ≥G2 AST), and the results were expressed as odds ratios (ORs).

In a sensitivity analysis, fully adjusted models were further adjusted for chemotherapy treatment (yes or no) and weeks of radiotherapy (3 or 5 weeks) to examine the robustness of associations.

Total energy (E) results were presented for a 100 kcal/day increment. The dietary carbohydrates and protein results were presented for 30 g/day increments, and dietary fat was presented for 15 g/day increments. Further, protein and carbohydrate subcomponents were presented for 15 g/day increments, whereas fat subcomponents were presented for 5 g/day increments. Finally, the results of the %E derived from carbohydrates, proteins, and fat were presented for a 1% increment. Figure 1 lists the missing data on covariates. To maximize data availability, missing data were handled using a regression-based imputation method (single imputation) with the PROC MI procedure in SAS. Statistical tests were two-sided, and a 95% confidence interval (CI) was set for the analyses. Data analyses were generated using STATA/SE software, version 18.0 (StataCorp, College Station, TX, USA), and SAS/STAT software, Version 9.4.

## 3. Results

### 3.1. Characteristics of the ATHENA Participants Included in the Analysis

The current study comprised 161 women with a mean age of 57 years (SD 10.3). Characteristics of the ATHENA participants are reported in Table 1. Of the 161 participants, 27% (n = 43) reported AST ≥ G2, and the rest of the sample (73%, n = 118) were classed as AST grade 0 or 1. Hypertension was observed in 28.5% (n = 46) of the participants. Further, 43.4% (n = 70) of participants were classed as overweight and 16.1% (n = 26) as obese based on their BMI (kg/m^2^), and 98% (n = 158) reported the use of prescribed hormone therapy, of which 42% (n = 69) was letrozole. The mean total energy intake per day of the participants was 1731 kcal (SD 463) (Table 2). At T0, the mean total carbohydrate, protein, and fat intakes per day were 211 (SD 66), 73 (SD 18), and 69 (SD 21) grams, respectively. The mean %E from carbohydrates was 48% (SD 2), from protein was 17% (SD 7), and from fat was 35% (SD 5).

### 3.2. Relationship Between Dietary Macronutrient Intakes and AST

The fully adjusted macronutrient models (Model 2) (refer to Table 3) indicated that a higher intake of dietary carbohydrates (per 30 g/d) was associated with a 30% higher odds of ≥G2 AST post-radiotherapy among BC patients (OR = 1.30; 95% CI 1.01 to 1.67; *p* = 0.038). In contrast, a higher intake of dietary protein (per 30 g/d) was inversely associated with a 76% lower odds of ≥G2 AST post-radiotherapy (OR = 0.24; 95% CI 0.06 to 0.91; *p* = 0.036). Finally, total energy intake (per 100 kcal/day) or dietary fat (per 15 g/d) were not associated with ≥G2 AST post-radiotherapy in the fully adjusted model (Table 3).

### 3.3. Relationship Between the Percentage of Energy Derived from Dietary Macronutrients and AST

Amongst %E models, in model 2 (Table 3), per unit, only higher %E from protein was inversely associated with a lower odds of ≥G2 AST by 22% (0.78; 95% CI 0.65 to 0.94; *p* = 0.010). However, there was no evidence of an association between dietary carbohydrates or fat (%E forms) and the odds of ≥G2 AST post-radiotherapy.

### 3.4. Relationship Between Dietary Macronutrient Subcomponent Intakes and AST

Fully adjusted macronutrient subcomponent models showed that a higher increment of animal protein (per 15 g/d) was inversely associated with a lower odds of ≥G2 AST by 51% (0.49; 95% CI: 0.25 to 0.95; *p* = 0.035) (refer to Table 4). No association was found for either dietary carbohydrates or fat subcomponents.

In the sensitivity analyses, all the results remained statistically significant after additional adjustments for chemotherapy treatment and radiotherapy duration (3 or 5 weeks) (refer to Table 5 and Table 6).

## 4. Discussion

The overall findings in the present study showed that dietary macronutrient intakes could impact the AST risk differently in BC women post-radiotherapy. Dietary carbohydrates were associated with higher, whereas dietary protein and its animal protein subcomponents were associated with a lower risk of ≥G2 AST among BC women undergoing radiotherapy. However, there was no evidence of an association with dietary fat and its subcomponents.

To our knowledge, this is the first prospective study to assess the relationship between diet, specifically the macronutrient composition, and skin toxicity (≥G2 AST) in women with BC undergoing radiotherapy. Concerning the diet, one study suggested a radioprotective effect of wine consumption (1–2 glasses/d) on skin toxicity among BC women post-radiotherapy [26]. Another study analyzed the effect of anthocyanin supplementation derived from purple corn cobs on AST in BC women undergoing radiotherapy [18]. However, previously, other studies investigated various radiation-induced toxicities apart from skin-related ones in multiple organs, such as the lung, central nervous system, head and neck, and gastrointestinal system [27]. To elaborate, an RCT suggested that a high-fiber diet might lower gastrointestinal toxicity in pelvic radiotherapy [28]. Another meta-analysis [29] found a positive impact of enteral feeding (tube feed) in head and neck cancer.

Our findings showed that per-day increments of 30 g of dietary protein and 15 g of animal protein (for instance, fish, seafood, poultry, and animal meat) were associated with a lower risk of ≥G2 AST in women with BC. Since proteins are the main building blocks, they are responsible for cell renewal, tissue growth, and repair for wound healing. Proteins are involved in the wound healing process in the following ways: DNA and RNA formation, inflammation reduction, immunity function, collagen and elastin formation, epidermal growth, and keratinization of the wound site [30,31,32]. These findings might indicate that choosing the right amount and type of proteins will help in the remodeling phase to lower ≥G2 AST, including tender or bright erythema, patchy moist desquamation/moderate edema, ulceration, hemorrhage, and necrosis at the radiated site of the breast. The remodeling phase involves protein in the form of collagen deposition in a well-formed network coupled with fibrin and calcium. The dietary supply of collagen, elastin, and fibrin is found in animal protein sources, such as poultry, fish, seafood, and animal meat [33,34].

Regarding our study’s dietary animal protein findings, a study showed that high adherence to an animal dietary pattern increased inflammation and dermatitis (a similar manifestation observed in G1 AST post-radiation) but in skin cancer [35]. Further studies are required to explore ≥G2 AST in BC. Nevertheless, cautious care must be taken concerning animal protein amounts and choices because red/processed meat has previously been linked to a higher risk of BC [36]. Overall, these dietary protein-related findings might be helpful for clinical nutrition practice, reducing radiation-induced ≥G2 AST, and increasing the treatment response and overall immunity.

Our study’s carbohydrate findings for g/d indicated that controlling their amounts could be beneficial during radiation for lowering ≥G2 AST. However, %E carbohydrate findings showed no association and might be because they were not mutually adjusted for other %E macronutrients, unlike the g/d models. Moreover, there is a lack of nutritional science evidence and guidelines—well-designed studies are needed to evaluate an ideal %E from carbohydrates while mutually balancing other %E from proteins and fats optimal for consumption during BC radiation. Nevertheless, the following plausible mechanisms might explain the beneficial impact of controlling carbohydrate intakes: (1) controlling carbohydrates could metabolically starve cancer cells or force them to utilize oxidative phosphorylation instead of glycolysis or glycogenolysis [37]; (2) the mechanisms that can reduce cancer-treatment-related toxicities may be explained by the Warburg effect—cancer cells utilize glycolysis over oxidative phosphorylation, potentially preventing oxidative damage caused by reactive oxygen species [38]; and (3) increased oxidative stress in cancer cells could then sensitize them to chemotherapy and radiotherapy more so than normal cells, thereby reducing the dosages necessary for treatment and consequently reducing treatment-related toxicities. Also, findings showed no association between carbohydrate subcomponents and the risk of post-radiation ≥G2 AST in BC. However, the subcomponents comprised polysaccharides, and the CI was wide. Perhaps further studies could explore mono-, di-, and oligosaccharides to investigate plausible mechanisms and associations. Other studies have found beneficial effects of dietary interventions that used caloric-restriction diets, intermittent fasting, and ketogenic diets by lowering the per day carbohydrate intake as dietary strategies for patients undergoing radiation combined with chemotherapy [39,40]. However, these methods remain debated with inconsistent evidence and significant safety/ethical concerns [41,42].

Our findings did not show an association between dietary fat and its subcomponents and the ≥G2 radiation-induced AST risk. However, an animal study suggested that a high-fat diet potentially lowered the risk of AST in rats and postulated that skin lipids played an essential role in protecting against radiation beams [17]. However, our study did not observe this finding, possibly due to the contribution of several patient-related characteristics, including age, smoking, alcohol intake, and medications, on lipid metabolism during BC radiotherapy/chemotherapy [43,44]. Further, dietary fat subcomponents, including PUFAs and MUFAs, are typically consumed in smaller amounts, making it difficult to detect associations in the analysis.

There has been a limited focus on evidence-based dietary guidelines for macronutrient intakes during radiotherapy in BC. For instance, the American Cancer Society [45] published nutrition and physical activity guidelines for all types of cancer, including BC patients undergoing treatment, in 2007. However, one review critiqued these guidelines and recommended macronutrient guidelines to manage side effects and improve recovery during chemotherapy but not radiotherapy [40]. This highlights the need for recent evidence from observational studies that could contribute to making clinical nutrition guidelines for the entire spectrum, from BC diagnosis to the radiotherapy treatment response, with guidelines specific for reducing radiation-induced skin toxicity.

Our prospective study findings contribute to the evidence required to focus on radiation-induced ≥G2 AST during BC treatment, which could be significant in the oncology nutrition context. Moreover, prioritizing the macronutrient composition based on the type of treatment (radiotherapy alone or coupled with chemotherapy) in clinical settings could be a window of opportunity as patients have high compliance with dietary counseling while receiving treatment [46,47].

### Strengths and Limitations

There are several strengths to be considered. The findings were from a prospective study design. We had access to the dietary data and used international guidelines to classify AST readings to make the findings widely applicable and replicable in clinical settings. The regression analyses were adjusted for covariates useful in clinical applications [24,25]. The analyses were adjusted for anthocyanin treatment/placebo, thus accounting for the original study design [18]. The analysis findings were robust when adjusted for commonly used cancer treatments like chemotherapy. We used realistic macronutrient and subcomponent increments to provide meaningful interpretations required for clinical nutrition practice. There were some limitations to be considered. First, the study had a relatively modest sample size (n= 161), and this may affect the accuracy and generalizability of the research results. Second, the prospective findings captured relatively shorter time points between baseline and radiotherapy at T1 (after 3 or 5 weeks). Third, no recommendations/guidelines for dietary macronutrient intakes were available for a population with BC undergoing radiotherapy, unlike the general population, making it challenging for a comparison and clinical interpretation. Finally, self-reported dietary data were used, which might be prone to recall bias where the participants could have under/misreported dietary intakes impacting overall energy estimations [48,49,50].

## 5. Conclusions

These findings show that habitual dietary macronutrient intakes could impact acute skin toxicity differently among BC patients who underwent radiotherapy. Dietary carbohydrates were associated with a higher risk, whereas dietary protein and its animal protein subcomponent were associated with a lower risk of ≥G2 AST post-radiotherapy among BC patients. Cautiously balancing carbohydrates and protein intakes might be suggested for clinical nutrition management strategies for a ≥G2 AST reduction post-radiotherapy among BC women.

## Figures and Tables

**Table 1:** Baseline characteristics of the participants in the ATHENA trial (n = 161).

Variables	Mean or Frequency
N of participants (%)	161 (100)
Age, years (mean, SD)	57.0 (10.3)
Educational level, n (%)	
None	3 (1.8)
Up to lower secondary	34 (21.1)
High school	45 (28.0)
Upto postgraduate	27 (16.8)
Do not know	52 (32.3)
Body mass index, kg/m^2^ (mean, SD)	26.4 (4.4)
Body mass index (BMI) (kg/m^2^), n (%)	
18.5–24.9 (normal)	65 (40.3)
25–30 (overweight)	70 (43.6)
>30 (obese)	26 (16.1)
Co-morbidities, Yes, n (%)	
Hypertension	46 (28.5)
Diabetes Mellitus	8 (5)
Physical activity levels, Yes, n (%)	
Moderate intensity	21 (13)
Vigorous intensity	7 (4)
Smoking status, n (%)	
Yes	28 (17)
No	106 (66)
Former	27 (17)
Use of hormone therapy, Yes, n (%)	158 (98)
Use of letrozole, Yes, n (%)	69 (42)
Chemotherapy, Yes, n (%)	77 (48)
Antocyanin treatment, Yes, n (%)	83 (52)
Acute skin toxicity, n (%)	
Grade 0	49 (30)
Grade 1	67 (42)
Grade 2	43 (27)
Grade 3	2 (1)
Duration of radiotherapy, n (%)	
3 weeks	56 (35)
5 Weeks	105 (65)
Menopause, Yes, n (%)	100 (62)

**Table 2:** Nutrient intakes in the ATHENA project (mean values and standard deviations) (n = 161).

Nutrient Intakes ^a^	Athena Project Participants (n = 161)		
Mean Macronutrient Intake	Unit/Day	Mean	SD
Total energy	Kcal	1731	463
Total carbohydrate	G	211	66
Total protein	G	73	18
Total fat	G	69	21
%E (carbohydrate)	%	48	2
%E (protein)	%	17	7
%E (fat)	%	35	5
Starch	G	129	44
Total dietary fiber	G	18	5
MUFA	G	33	10
PUFA	G	7	2
SFA	G	25	9
Alcohol	G	4	7
Mean micronutrient intake			
Iron	Mg	11	3
Calcium	Mg	851	299
Sodium	Mg	1809	667
Potassium	Mg	2600	653
Zinc	Mg	10	3
Vitamin C	Mg	114	43
Vitamin B6	µg	2	0.39
Folate	µg	230	61

^a^ Dietary data presented were recorded at baseline (T0) and computed from the EPIMED FFQ.

**Table 3:** Association between macronutrient intakes at baseline (T0) and percentage of energy (%E) from macronutrients (g), and the odds of acute skin toxicity (AST) in ATHENA project (n = 161).

Macronutrient * Intake (Per Day Increment) n = 161	Odds of Skin Toxicity, Model 1			Odds of Skin Toxicity, Model 2		
**At Baseline (T0)**	**OR ^a^**	**95% CI**	***p* Value**	**OR ^b^**	**95% CI**	***p* Value**
Total energy (100 kcal)	1.09	1.01 to 1.18	0.022	0.93	0.85 to 1.03	0.18
Total carbohydrate (30 g)	1.31	1.04 to 1.66	0.019	1.30	1.01 to 1.67	0.038
Total protein (30 g)	0.33	0.09 to 1.16	0.085	0.24	0.06 to 0.91	0.036
Total fat (15 g)	1.43	0.87 to 2.36	0.15	1.52	0.89 to 2.59	0.12
**Macronutrient intake** **(%E increment) n = 161**	**Odds of skin toxicity, Model 1**			**Odds of skin toxicity, Model 2**		
**At baseline (T0)**	**OR ^c^**	**95% CI**	***p* Value**	**OR ^b^**	**95% CI**	***p* Value**
%E (carbohydrate)	1.04	0.98 to 1.09	0.15	1.03	0.97 to 1.10	0.21
%E (protein)	0.79	0.66 to 0.94	0.010	0.78	0.65 to 0.94	0.010
%E (fat)	0.97	0.90 to 1.04	0.44	0.96	0.89 to 1.04	0.42

OR, odds ratio; CI, confidence interval; CRP, C-reactive protein. * Mutually adjusted for other energy-contributing macronutrients. ^a^ Adjusted for age, body mass index, and treatment classification (treatment/placebo). ^b^ Additionally adjusted for hypertension, alcohol consumption (never/low, moderate and high intakes), smoking habits (yes or no), CRP levels at baseline, and letrozole medication. ^c^ Separate analysis between each %E dietary macronutrient and acute skin toxicity adjusted for age, body mass index, and treatment classification (treatment/placebo).

**Table 4:** Association between macronutrient subcomponents at baseline (T0), and the odds of AST in the ATHENA project (n = 161).

Macronutrient Subcomponents (g/d Increment) n = 161	Odds of Skin Toxicity, Model 1			Odds of Skin Toxicity, Model 2		
	OR ^a^	95% CI	*p* Value	OR ^b^	95% CI	*p* Value
Source of total carbohydrate †‡						
Starch (15 g)	1.13	0.94 to 1.34	0.16	1.14	0.94 to 1.37	0.16
Dietary fiber (15 g)	1.68	0.36 to 7.79	0.50	1.99	0.36 to 10.95	0.42
Soluble carbohydrate (15 g)	1.10	0.89 to 1.35	0.34	1.04	0.82 to 1.33	0.70
Sources of total fat †*						
SFA (5 g)	1.19	0.75 to 1.88	0.45	1.31	0.79 to 2.18	0.28
MUFA (5 g)	0.81	0.48 to 1.37	0.44	0.80	0.47 to 1.37	0.43
PUFA (5 g)	6.44	0.59 to 69.38	0.12	6.64	0.59 to 73.9	0.12
Sources of total protein *‡						
Animal protein (15 g)	0.56	0.29 to 1.07	0.082	0.49	0.25 to 0.95	0.035
Vegetable protein (15 g)	0.75	0.11 to 4.79	0.76	0.85	0.10 to 7.16	0.88

OR, odds ratio; CI, confidence interval. ^a^ Adjusted for age, body mass index, and treatment classification (treatment/placebo). ^b^ Additionally adjusted for hypertension, alcohol consumption (never/low, moderate and high intakes), smoking habits (yes or no), CRP levels at baseline, and letrozole medication. ‡ Adjusted for dietary fat intakes. † Adjusted for dietary protein intakes. * Adjusted for carbohydrate intakes. SFAs, saturated fatty acids; MUFAs, monounsaturated fatty acids; PUFAs, polyunsaturated fatty acids.

**Table 5:** Sensitivity analysis: association between macronutrient intakes at baseline (T0) and percentage of energy (%E) from macronutrients (g), and the odds of AST in the ATHENA project (n = 161).

Macronutrient * Intake (Per Day Increment) n = 161	Odds of Skin Toxicity		
	**OR ^a^**	**95% CI**	***p* Value**
Total energy (100 kcal)	1.07	0.98 to 1.16	0.10
Total carbohydrate (30 g)	1.29	1.00 to 1.67	0.046
Total protein (30 g)	0.23	0.06 to 0.89	0.034
Total fat (15 g)	1.56	0.91 to 2.70	0.10
**Macronutrient intake** **(%E increment)** **n = 161**	**Odds of Skin Toxicity**		
	**OR ^a,b^**	**95% CI**	***p* Value**
%E (carbohydrate)	1.03	0.97 to 1.10	0.20
%E (protein)	0.77	0.64 to 0.93	0.009
%E (fat)	0.96	0.89 to 1.05	0.43

OR, odds ratio; CI, confidence interval. * Mutually adjusted for other energy-contributing macronutrients. ^a^ Adjusted for age, body mass index, treatment classification (treatment/placebo), hypertension, alcohol consumption (never/low, moderate and high intakes), smoking habits (yes or no), CRP levels at baseline, prescribed hormone medication (letrozole), duration of radiotherapy (3 or 5 weeks), and chemotherapy treatment (yes or no). ^b^ Separate analysis between each %E dietary macronutrient and acute skin toxicity adjusted for age, body mass index, and treatment classification (treatment/placebo).

**Table 6:** Sensitivity analysis: association between macronutrient subcomponents at baseline (T0), and the odds of AST in the ATHENA project (n = 161).

Macronutrient Subcomponents (g/d Increment) n = 161	Odds of Skin Toxicity		
	**OR ^a^**	**95% CI**	***p* Value**
Source of total carbohydrate †‡			
Starch (15 g)	1.14	0.94 to 1.37	0.16
Dietary fiber (15 g)	1.99	0.36 to 10.95	0.42
Soluble carbohydrate (15 g)	1.04	0.82 to 1.33	0.70
Sources of total fat †*			
SFA (5 g)	1.31	0.79 to 2.18	0.28
MUFA (5 g)	0.80	0.47 to 1.37	0.43
PUFA (5 g)	6.64	0.59 to 73.9	0.12
Sources of total protein *‡			
Animal protein (15 g)	0.47	0.23 to 0.92	0.030
Vegetable protein (15 g)	0.85	0.10 to 7.16	0.88

OR, odds ratio; CI, confidence interval. ^a^ Adjusted for age, body mass index, treatment classification (treatment/placebo), hypertension, alcohol consumption (never/low, moderate and high intakes), smoking habits (yes or no), CRP levels at baseline, letrozole medication, duration of radiotherapy (3 or 5 weeks), and chemotherapy treatment (yes or no). ‡ Adjusted for dietary fat intakes. † Adjusted for dietary protein intakes. * Adjusted for carbohydrate intakes. SFAs, saturated fatty acids; MUFAs, monounsaturated fatty acids; PUFAs, polyunsaturated fatty acids.

## Data Availability

The data underlying this article will be shared on reasonable request by the corresponding author. The data are stored in an institutional repository (https://repository.neuromed.it, accessed on 28 December 2024), and access is restricted by the ethical approvals and the legislation of the European Union. Data described in the manuscript, code book, and analytic code will be made available upon request pending application and approval by the EU-ATHENA trial Steering Committee.

## Appendix A

EU-ATHENA Trial Investigators: Steering Committee: Maria Benedetta Donati (chairperson) ^1^, Chiara Tonelli ^2^, Chiara Cerletti ^1^, Licia Iacoviello ^1,3^, Alessio Giuseppe Morganti ^4^, Giovanni de Gaetano ^1^.

Anthocyanin Supplement Development: Chiara Tonelli ^2^, Katia Petroni ^2^.

Patient Recruitment: Francesca Bracone (Coordinator) ^1^, Amalia De Curtis ^1^, Livia Rago ^5^, Francesco Deodato ^6,7^, Mariangela Boccardi ^7^, Gabriella Macchia ^7^, Cinzia Digesù ^7,^*, Savino Cilla ^8^.

Statistical Analyses: Augusto Di Castelnuovo ^1^.

Present Affiliations:

1 Research Unit of Epidemiology and Prevention, IRCCS Neuromed, Pozzilli, Italy.
2 Department of Biosciences, Università degli Studi di Milano, Milano, Italy.
3 Department of Medicine and Surgery, LUM University “Giuseppe Degennaro”, Strada Statale 100, Km 18, 70010 Casamassima (BA)—Italy.
4 Department of Experimental, Diagnostic and Specialty Medicine (DIMES), University of Bologna, Bologna.
5 Associazione Cuore Sano ETS, Campobasso.
6 Istituto di Radiologia: Università Cattolica S. Cuore, Rome, Italy.
7 Radiotherapy Unit: Responsible Research Hospital, Campobasso, Italy.
8 Medical Physic Unit: Responsible Research Hospital, Campobasso, Italy.
* deceased.
